# Housing, opportunities, motivation and engagement (HOME) for homeless youth at-risk for opioid use disorder: study protocol for a randomized controlled trial

**DOI:** 10.1186/s13722-021-00237-7

**Published:** 2021-05-12

**Authors:** Natasha Slesnick, Laura Chavez, Alicia Bunger, Ruri Famelia, Jodi Ford, Xin Feng, Sarah Higgins, Eugene Holowacz, Soren Jaderlund, Ellison Luthy, Allen Mallory, Jared Martin, Laura Walsh, Tansel Yilmazer, Kelly Kelleher

**Affiliations:** 1 Department of Human Sciences, College of Education and Human Ecology, The Ohio State University, 1787 Neil Ave, Columbus, OH 43210 USA; 2 Center for Innovation in Pediatric Practice, The Abigail Wexner Research Institute At Nationwide Children’s Hospital, 700 Children’s Drive, Columbus, OH 43205 USA; 3 College of Social Work, The Ohio State University, 1947 N. College Road, Columbus, OH 43210 USA; 4 College of Nursing, The Ohio State University, 1585 Neil Avenue, Columbus, OH 43210 USA

**Keywords:** Homelessness, Opioid use disorder, Prevention, Housing First

## Abstract

**Background:**

Homeless youth experience high rates of substance use disorders, exposures to violence, mental and physical health conditions, and mortality. They have been particularly affected by the opioid crisis. However, no study to date has used a randomized controlled design to test preventive interventions of opioid and other drug use among this vulnerable population. Resolution of youth homelessness through housing and supportive services including prevention services, often referred to as “Housing First,” has great potential to reduce the likelihood for the development of an opioid use disorder as well as other problem behaviors associated with living on the streets. Housing First has been tested through randomized trials among homeless adults with mental health and substance use disorders, but has not been empirically tested for opioid prevention among homeless youth.

**Methods:**

Homeless youth will be recruited from a drop-in shelter site frequented by disconnected youth; they will be screened for eligibility, including current homelessness, age 18–24 years, and not currently meeting criteria for opioid use disorder (OUD). In a controlled trial, 240 youth will then be randomized to one of two conditions, (1) housing + opioid and related risk prevention services, or (2) opioid and related risk prevention services alone. This project utilizes existing efficacious models of prevention to address opioid-related risks, including motivational interviewing, strengths-based outreach and advocacy, and an HIV risk preventive intervention. Follow-up will be conducted at 3, 6, 9 and 12-months post-baseline. The economic cost of each intervention will be determined to support implementation decisions with other providers and their funders.

**Discussion:**

This study will provide essential information for researchers and providers on the efficacy of housing + opioid and related risk prevention services in an RCT for effects on opioid use and mechanisms underlying change. Because youth experiencing homelessness are at increased risk for a variety of adverse outcomes, the proposed intervention may produce substantial health care benefits to the youths and society at large.

*Trial registration* ClinicalTrials.gov, NCT04135703, Registered October 13, 2019, https://clinicaltrials.gov/ct2/show/NCT04135703?term=NCT04135703&draw=2&rank=1#contacts

## Background

The rapid increase in opioid use among youth is the most alarming drug use trend in the US [1, 2]. While opioid use in the general population received significant media and scholarly attention in recent years, very little experimental research exists on opioid use among homeless or street-involved youth. However, homeless youth are at high risk for opioid use and transition to injection drug use [3, 4]. Given the high rates of opioid use as well as health risks and mortality in homeless youth, it is surprising that no study to date utilizes a randomized controlled design to test a preventive intervention for opioid use. In fact, empirical evaluations of preventive interventions for homeless youth are essentially non-existent [5]. Similarly, little is known about subgroups of homeless youth, but sexual, gender and racial minority youth are overrepresented among homeless youth populations [6], and experience unique challenges.

This randomized trial, entitled HOME: housing, opportunities, motivation and engagement will provide essential information for researchers and providers on the efficacy of housing + prevention services in an RCT focused on the prevention of opioid use among youth experiencing homelessness between the ages of 18 to 24 years. In this paper, we justify and describe the methods and protocol used to evaluate the HOME intervention with these at-risk youth.

## Need for preventive interventions

Youth experiencing homelessness carry a disproportionate burden in the opioid use epidemic. For example, 79% of a Toronto-based sample of homeless youth reported injection drug use and attempts to quit heroin use, and 52% reported a non-fatal opioid overdose [3]. Over half of a sample of homeless youth in Los Angeles reported prescription drug misuse, and 21% reported current misuse, with opioids identified as the most commonly misused drug [7]. Data suggest a progression in severity of use, highlighting the importance of preventive intervention efforts for those who begin opioid use. In particular, using the “At-Risk Youth Study” data, which included longitudinal data on street-involved youth aged 14–26 in Vancouver, 21% of the youth who reported non-medical use of prescription opioids (codeine, oxycontin, and morphine) at the beginning of the study transitioned to injection drug use by 11.2 months [2]. Additional research using the “At-Risk Youth Study” data found that non-prescription opioid misusers were more likely to transition to binge drug use, daily heroin use and were more likely to experience violence compared to their homeless peers who did not misuse prescription opioids [8]. Male sex was more highly associated with making a switch from non-medical prescription drug use to other illegal drug use, and more than 45% of the sample experienced a non-fatal overdose [4, 9]. Youth aging out of foster care are another at-risk subgroup who experience heightened risk for homelessness, face challenges accessing services and are at increased risk for poor health outcomes including drug use [10]. Homeless youth report barriers to seeking interventions including a lack of housing, finances, access to services, motivation, intervention options, but at the same time they have easy access to heroin and high levels of stress [3]. Taken together, studies underscore the significant risk for opioid misuse among youth experiencing homelessness and highlight the importance of identifying interventions that can curb the epidemic among this high-risk group of young people.

Social exclusion from services in particular, or society in general, may contribute to the poor health outcomes among youth experiencing homelessness [11]. Goering et al. argue that inadequate access to services, housing, employment, weak social capital and restricted access to safety measures (seeking police assistance, medical assistance) underlie homeless youth’s disparity in victimization and health outcomes [12]. As noted by Goering et al. (1997), social exclusion is “the process of being shut out, fully or partially, from any of the social, economic, political or cultural systems which determine the social integration of a person in society. Social exclusion may, therefore, be seen as the denial of civil, political and social rights of citizenship” [13]. Homeless adults with access to a social service worker, or who utilize community services, are more likely to exit homelessness [14, 15]. One study showed that the more connections youth had with formal and informal social systems at the beginning of the study, the more likely they were to decrease the number of homeless days and to start with fewer homeless days [16].

## Prior prevention interventions

Opioid and other drug prevention research advanced considerably over the past 20 years. Improvements in decreasing availability of drug supply, bolstering family and school education around drug use among young people, and interventions in home and school settings all have shown effectiveness in preventing progression of youth drug abuse [17–19]. In part, these advances in preventive services delivered in home and school settings reflect the importance of social and environmental influences on drug use by youths. It is exactly these same influences which challenge preventive interventions for youth experiencing homelessness. Successful interventions for families are not relevant when a young person is separated from family for safety, emotional, financial or other reasons and is unwilling or unable to return. Similarly, school interventions are not relevant for those living on the streets for the most part. The circumstances for each young person on the street may vary nightly and locations may change frequently. In short, delivery of preventive interventions to street-living youth is especially challenging and may be one of the reasons that youth experiencing homelessness have high rates of opioid use disorder. Moreover, traditional housing services often require that youth with mental disorders or drug use demonstrate a period of sustained treatment or abstinence before receiving supportive housing assistance.

According to Aidala et al., homelessness is a source of chronic stress where the focus on shelter and survival supersedes efforts to reduce risk [20]. Substance use can be a response to stress, and/or untreated mental health conditions. In fact, considerable evidence exists that when core needs like shelter are not met, a state of deprivation cognition or excessive attention to immediate relief impedes the learning of new skills, self-efficacy and other prevention targets [5, 21]. Aidala et al. documented the change in risk behaviors associated with changes in housing status among homeless HIV positive adults [20]. The odds of recent hard drug use were four times higher among those who remained homeless compared to those that obtained stable housing. Those who obtained housing were half as likely to use hard drugs and needles, to share needles, and engage in unprotected sex as those whose housing situation did not change. They concluded that housing should be included as an important tool in the positive prevention arsenal, and “housing as healthcare” holds great promise [20]. Although housing is expensive and challenging, the costs of the opioid epidemic and homelessness are greater. Indeed, research has found that US taxpayer costs of providing supportive housing are almost 50% lower than the costs of homelessness [22]. Given that the opioid epidemic has an estimated economic burden of $78.5 billion in the US, and the high prevalence of opioid use among homeless populations, it is likely that providing housing would also have a cost-saving effect on the financial impact of the opioid epidemic [23]. Through this study, we seek to test whether a replicable housing intervention leads to a reduction in risk of opioid use and other risk behaviors.

Housing First/Pathways to Housing is a newer approach that accepts shelter/housing as a basic right and the first step towards successful interventions of any type [24–27]. Housing First proposes that: (i) individuals be placed into longer term housing as soon as possible, (ii) client strengths and choices direct initial supportive services offered, (iii) shared accountability for rent, utilities and participation are encouraged, and (iv) individualized prevention and treatment interventions for health, mental health and drug use are incorporated. Evidence from randomized trials in the US, Canada and Europe supports the use of Housing First models to improve outcomes for adults with mental disorders and drug use disorders with improvements found in patient outcomes, costs, crime and homelessness [28–31]. Gaetz and Dej suggested that with appropriate modifications the Housing First model could be even more effective for youth experiencing homelessness who may have less severe cognitive limitations, drug use at an earlier stage, and be more open to early intervention [24]. Gaetz et al. suggested five core principles for a Housing First model to address youth homelessness and the epidemics of mental disorders and drug use in this population [24, 32]. The five principles are: (a) immediate access to permanent housing without performance preconditions, (b) positive youth development orientation, (c) self-determination with coaching, (d) individualized supports and (e) community integration and socialization.

## The present study

The overarching goal of the present study is to evaluate a comprehensive intervention for the prevention of opioid use disorder and for increasing other resilient outcomes in young adults experiencing homelessness. The primary aim is to evaluate the relative efficacy of housing + opioid and related risk prevention services compared to prevention services alone (N = 240). We hypothesize that youth receiving the 6-month housing + prevention services will have more positive 3, 6, 9 and 12-month outcomes than those receiving prevention services alone. Further, we hypothesize that fewer Housing First youth will progress to OUD and will be more likely to remain housed at one year. A secondary aim is to test the effects of the primary and secondary mediators (primary: service connections, social support; secondary: stress, self-efficacy) on the primary outcome (opioid use/time to OUD) and secondary outcomes. We hypothesize that inasmuch as the intervention triggers successful social micro- and meso-system interactions or social resources (e.g., advocate and social service meetings), individual resources will be activated—e.g., stress will decrease and self-efficacy will increase, leading to multiple positive outcomes.

## Methods/design

### Study design

The study is a randomized controlled trial, whereby 240 youth will be randomized into either housing + opioid and related risk prevention services (n = 120), or opioid and related risk prevention services alone (n = 120). In this study, we seek to determine if adding housing to opioid and related risk prevention services yields a significant benefit above and beyond the benefit yielded by opioid and related risk prevention services alone. Follow-up assessments to collect study outcomes will be conducted at 3, 6, 9 and 12-months post-baseline to evaluate stability of effects.

### Participants

Youth will be eligible to enroll in the study if they meet the following criteria: (1) youth is between the ages of 18 to 24 years; (2) youth meets the criteria for homelessness as defined by the federal McKinney-Vento Act as “lacking a fixed, regular, stable, and adequate nighttime residence” and includes “living in a publicly or privately operated shelter designed to provide temporary living accommodations, or a public or private place not designed for, or ordinarily used as, regular sleeping accommodations for human beings” [33]; and (3) youth does not meet diagnostic criteria for OUD as assess by the Structural Clinical Interview for DSM-5 (SCID) [34]. Lifetime opioid use is not an exclusionary criterion; however, youth cannot meet diagnostic criteria for OUD at the time of the baseline assessment. The age range was selected because it has been the study team’s experience that landlords will not accept leases signed by youth under the age of 18 years. In addition, “homeless youth” commonly refers to those up to the age of 24 years, and generally reflects the age range of the homeless youth population served by providers across the country [35].

### Community engagement

The study will employ a Community Advisory Group (CAG) of key stakeholders including representatives of homeless youth, homeless providers, landlords, substance use treatment experts and policy makers. The CAG will meet throughout the project period, with greater frequency at the beginning and gradually decreasing in frequency. At the beginning of the study, the CAG will review and provide input on study procedures, such as the protocol for identifying youth who are eligible for the study and providing input on any barriers that arise for recruiting landlords willing to rent to participants. This feedback is directly integrated into the study protocol. Moving forward, as the study begins recruitment and follow-up, the CAG will be informed of recruitment progress and challenges, providing suggestions and feedback as needed. Finally, as the study concludes the CAG will give input on best approaches for easing transition of intervention to practice. The CAG is an integral part of the design of the study in order to evaluate both the effectiveness and implementation determinants at an early stage. Too often, interventions do not reach those for whom the interventions were developed [36]. Engaging community stakeholders from the earliest phases of research, as such continuous involvement is critical to dissemination and uptake [36].

### Recruitment protocol

The study occurs in a large Midwestern city in the US. The city has one drop-in center for homeless youth between the ages of 14–24 years. Approximately 1000 unduplicated homeless youth are served annually, with 70–95% of homeless youth reporting problem substance use, similar to national samples of homeless youth [37]. The single adult shelters have a total of 457 beds for single men and 97 for single women on any given night. Service-connected youth will be recruited from the drop-in and adult shelters, non-service-connected youth will be recruited through outreach to local soup kitchens, sandwich lines, the streets, parks and libraries. Project staff maintain offices within a local drop-in center serving youth experiencing homelessness. Youth who are engaged on the streets will be transported to the drop-in center. A research assistant will engage and screen youth to determine basic eligibility for the study. After the brief screening and stated interest in the project, written consent will be obtained and the Structured Clinical Interview for DSM-5 Disorders (SCID) section on Opioid Use Disorder will be administered to determine formal eligibility [34]. Upon completion of the baseline assessment interview, youth will be randomly assigned to the intervention conditions using a computerized urn randomization program. At least four advocates will provide services so that advocate effects can be examined [38], and each advocate will provide all opioid and related risk prevention services to the youth on their caseload. For example, even with standardized training and protocols it is possible that unmeasured differences in the personal attributes of advocates could influence youth’s level of engagement with the interventions. Advocates will be crossed by condition to “equate” conditions on advocate characteristics. This approach is preferred over nesting, as advocates in both conditions are trained in the same procedures, which include strengths-based outreach and advocacy (SBOA), HIV prevention, and Motivational Interviewing (MI). Nesting would likely be considered the better choice if treatment philosophies differed among the intervention conditions. RA’s will be blind to condition at all assessment timepoints.

### Description of interventions

The HOME interventions integrate independent housing with opioid and related risk prevention services. Prevention services that will be delivered to both study groups include the following: SBOA, HIV prevention and MI [39] (Table 1). SBOA, HIV prevention and MI occur simultaneously in this approach. Each component of the intervention is described below in more detail. Youth who are randomized to the housing condition will receive housing assistance consistent with a Housing First approach. The housing assistance is not contingent on youth’s substance use or attendance of prevention services [19, 20]. To increase treatment participation, youth will receive a $5 McDonald’s, Burger King, Wendy’s, or other food gift card for every advocacy session they attend.

**Table 1:** Description of prevention interventions

Prevention interventions	Goals/Content	Duration and timing
Strengths-based outreach and advocacy	*Session 1* Review each of six general areas for youth’s needs and goals: (1) housing needs; (2) health care; (3) food; (4) legal issues, (5) employment and (6) education. An intervention plan will be guided by the initial review and the youth’s goals *Subsequent sessions* Continue to address progress towards meeting youth’s goals. There is no limit to number of sessions and frequency will depend on youth’s preferences. Advocates are available 24 h for crises	6 months. (No limit on number of contacts)
HIV prevention	*Session 1* Advocate will review AIDS education, assessment of risk, risk reduction and skills practice *Session 2* focuses on sexual assertiveness and practicing negotiation. Role plays are incorporated to allow the youth to practice social competency skills relevant to their life situations	2 sessions throughout the 6 months of intervention
Motivational interviewing	*Session 1* Advocate will conduct open-ended MI, to establish therapeutic rapport and elicit client change talk *Session 2* Advocate continues to focus on enhancing intrinsic motivation for change, developing discrepancy, transitioning as appropriate into the negotiation of a change plan and evoking commitment to the plan	2 sessions throughout the 6 months of intervention
Housing	Costs of housing, including security deposit, application fees, rent and utilities for six months are paid on behalf of participant directly to landlords and utility companies at the beginning of each month. Youth are also provided with furniture through the Furniture Bank of Central Ohio	6 months of housing assistance beginning upon youth signing lease

#### Strengths-based outreach and advocacy (SBOA)

Those individuals experiencing homelessness with access to a social service worker, or who utilize community services, are more likely to exit homelessness [14, 15, 40]. SBOA focuses on identifying and engaging youth from the streets and drop-ins/shelters etc. and assisting these youth to meet their basic needs (i.e., referrals to food pantries), obtain government entitlements (i.e., SSDI/SSI, cash assistance, food stamps), and connect to other needed supports (education, job training). The advocates provide referrals and/or transport youth to appointments as needed. The initial meeting provides an opportunity to gather information and follow-up meetings will assess progress towards meeting youth-directed goals and objectives (Table 1). Once this review is complete, an initial intervention plan is developed with specific goals and objectives.

#### HIV prevention

Every youth will receive the 2-session HIV prevention intervention which uses cognitive-behavioral techniques with a focus on skills building/behaviors (role plays with condom application, cleaning needles, communication/negotiation and problem solving), which has been used in prior projects with homeless youth with success reducing risk behaviors [41, 42]. Successful practice of skills is expected to increase confidence and self-efficacy, which is expected to increase the youth’s use of skills including condom use and negotiation in other micro-system interactions.

#### Motivational interviewing (MI)

Typically offered as a brief intervention of 1–2 sessions, Motivational Interviewing has a strong record of efficacy in the prevention and treatment of alcohol and other drug use disorders [39, 43]. MI assumes that the responsibility and capability for change lie within the client and needs to be evoked (rather than created or installed). Baer, Peterson and Wells provide some rationale for utilizing brief feedback and motivational intervention with street living youth—the intervention is less costly and demands much less of a hard-to-reach population than more intensive interventions [44]. Utilizing a sample of runaway adolescents recruited from a runaway shelter, Slesnick et al. found that substance use reductions were significant for those assigned to MI even two years post-treatment [45]. The advocate will administer MI. Five principles guide the practice of MI: express accurate empathy for the client with reflective listening, “develop discrepancy” between client’s goals and behaviors, avoid confrontation with the client, roll or adjust to resistance from the client, and support client’s self-efficacy [46]. The Project Match manual was adapted for homeless/runaway youth in prior trials in consultation with William R. Miller and Bo Miller (NIAAA grant no. R01AA12173 and NIDA grant R29DA11590). Adaptation of the manual included attention to the unique life situation of homeless youth in understanding motivations and challenges to recovery while homeless.

#### Housing

In the housing + opioid and related risk prevention services condition, youth will be housed in an apartment of their choosing and receive 6 months of utility and rental assistance of up to $600 per month. Our prior research has found that $600 is sufficient based on the local housing market rental prices to obtain housing for youth [47, 48]. The independent housing is not contingent on the youth’s substance use or attendance in prevention services [26, 27]. The advocate will work with the youth to identify appropriate housing among the available choices throughout the city and will initiate the procedure for payment from Nationwide Children’s Hospital directly to the landlord once housing is identified and the youth’s application is approved by the landlord. Landlords who oversee rental properties within the project’s rental budget are identified through advertisements and contacted directly to determine their willingness to rent units for the project prior to submitting applications for participants. The project will cover damage deposit, application fees (including the federal adjustment bureau (FABCO) credit report), and will automatically pay the landlords the rental checks at the beginning of each month. Nationwide Children’s Hospital will not sign leases on behalf of the youth, and so the youth will sign the lease. Youth will live alone or with their child dependents if applicable. As youth near the end of study-provided utility and rental assistance, the advocate will work with youth to prepare them for assuming responsibility for the housing costs, assisting them with linkage to employment and other community resources as needed. As is usually provided with SBOA, the advocate will assist youth in the comparison group (risk prevention services alone) with obtaining housing within the community, but they are not financially provided housing by the project. In addition, these youth are not provided with financial assistance at the conclusion of the study.

### Supervision and treatment adherence

The advocates are hired from the community and may include formerly homeless youth/adults or may include social workers or those from a similar field with experience working with those experiencing homelessness and who are comfortable engaging and working with individuals in non-traditional settings. Research assistants (RAs) include undergraduate/graduate students or staff who will collect data and will be blind to intervention condition. Advocates and the RAs will participate in a two-day training led by the first author on engagement, tracking and assessment procedures, and weekly supervision. Because of the range of backgrounds of advocates, the training will be tailored to each person’s unique needs and skills. Training will include in vivo observation and modeling by the first author to ensure that the outreach workers/advocates are comfortable identifying and engaging homeless youth. MI and HIV prevention training of advocates consists of readings and at least a two-day training including role play exercises and ongoing supervision. Advocates will continue with role play meetings beyond the two days until it is determined that they are both comfortable and competent with employing the proposed procedures. Adherence to the intervention procedures will be independently evaluated by two clinical graduate students through digital recordings of sessions. MI and SBOA fidelity measures have been used successfully to ensure adequate fidelity in prior trials. A random sample of 20% of sessions will be coded to ensure that the procedures are delivered as intended. Percent performance of prescribed behaviors and undesirable behaviors will be used as measures of adherence.

### Ethics and data monitoring

The Ohio State University’s (OSU) Institutional Review Board has reviewed and approved the research study. Adverse events will be monitored, and whether youths show deterioration as a function of the intervention received will be assessed (e.g., increase in substance use, mental health symptoms, etc.). Should a client experience a serious adverse event that was unanticipated and believed to be related to study procedures, the OSU IRB and NIDA will be notified within 48 h. Adverse events that are unexpected and related to the study, but not meeting the definition of a serious adverse event will be reported to the IRB within 10 days of the MPI’s discovery of the event. The OSU IRB reviews the adverse event report and determines if the event is a result of study procedures. If the event is considered a direct result of study procedures, the MPIs and the board will meet within 48 h and will discuss the modifications to the protocol that are needed in order to prevent future adverse events. A data safety monitoring board comprised of three members experienced in clinical trials and/or working with vulnerable populations will be convened twice yearly to review safety and progress across all research projects. If any intervention is found to cause harm to participants, the trial will be stopped. The board’s summary report, any recommended changes, and other required data reports on adverse event cases will be submitted to the IRB and our NIDA project officer.

### Protocol adaptations due to COVID-19

As a result of the COVID-19 pandemic, RAs’ and research subjects’ temperatures are taken prior to any face-to-face interaction, and all are provided with masks and hand sanitizer for use before and after in-person assessment at the drop-in center or other in-person meetings. Follow-up assessments and the majority of advocacy meetings are conducted by phone.

## Data collection

**Fig. 1:**
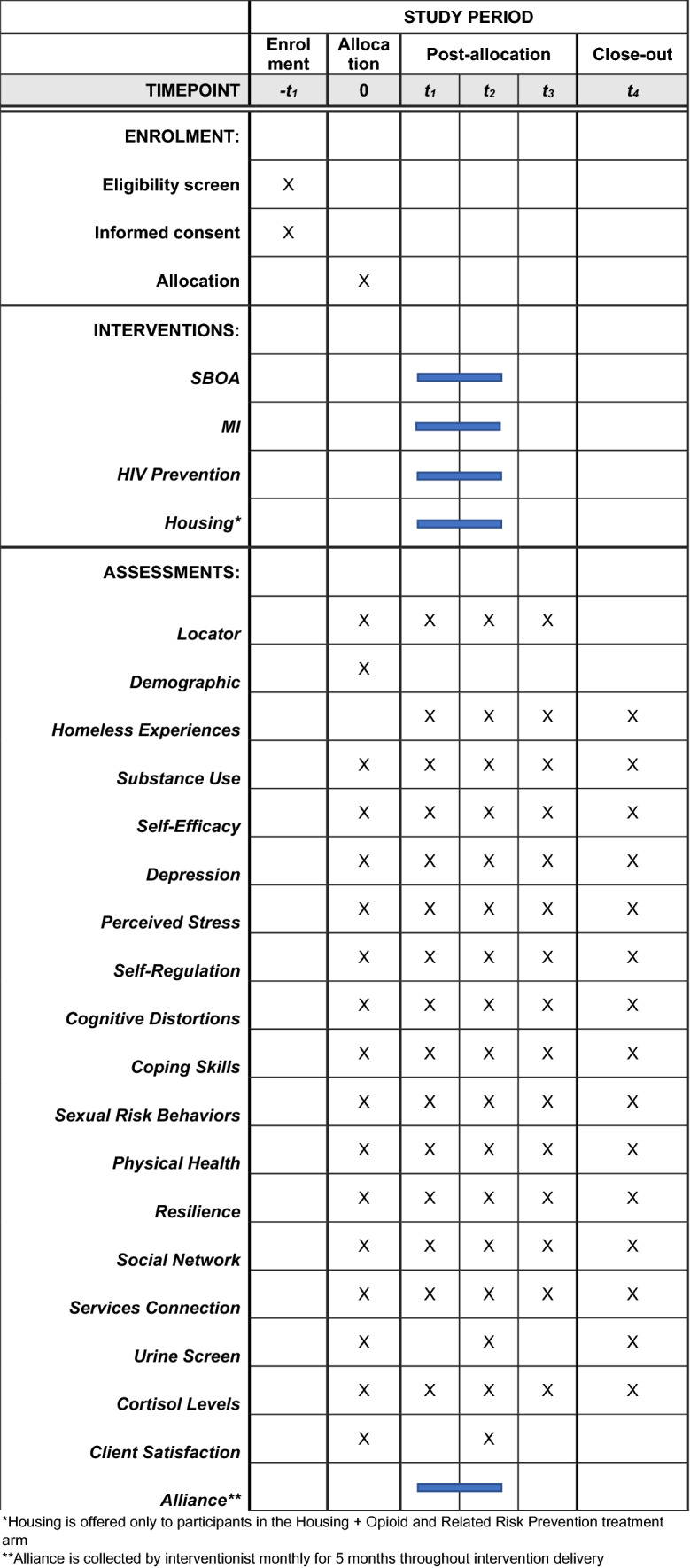
Example template of recommended content for the schedule of enrolment, interventions, and assessments.*Housing is offered only to participants in the Housing + Opioid and Related Risk Prevention treatment arm **Alliance is collected by interventionist monthly for 5 months throughout intervention delivery

The baseline and follow-up assessments will include self-report, interview, and physiological measures. At baseline a comprehensive interviewer-administered demographic questionnaire will be collected, as well as a homelessness experiences questionnaire (HEQ) used in prior studies [49]. These questionnaires will assess core variables used to characterize and compare samples. Assessments at baseline as well as follow-up time points (3, 6, 9, 12 months) will also collect primary and secondary outcome measures, and primary and secondary mediators (Fig. 1). The baseline and follow-up interviews will take approximately 2.5 h to complete each time. This assessment burden was found to be reasonable in our other projects. Youth will be offered frequent breaks and food and beverages to increase comfort. All participants will be assessed monthly during treatment via the Working Alliance Inventory (see below for description), receiving a $10 gift card each time. Youth will receive a $50 gift card at baseline and at each follow-up assessment. In order to maintain contact with youth and increase retention in data collection, extensive locator information will be obtained at the baseline assessment and follow-up assessments, in which youth designate hang-out spots in Columbus, as well as collateral contacts (friends or family members who may know where the youth is if project staff lost touch with them). Data are quality assured, double entered and electronically verified as they are collected. All hard copy data are stored in locked file cabinets in locked rooms within the drop-in center and/or university office. Electronic data are maintained on a password/duo mobile protected university network that employs a Cisco PIX Security Appliance as its firewall. Final trial results will not be directly communicated with trial participants; however, the results will be reported in peer-reviewed publications. A de-identified dataset will be shared with the sponsor and made publicly available.

### Primary outcomes

The main outcomes of the study are (1) time to OUD, and (2) percent days of opioid use in the past 90 days. The Structured Clinical Interview for DSM-5 Disorders (SCID) will be used to determine whether youth meet criteria for OUD at assessments. The SCID is a semi-structured diagnostic interview for DSM-5 diagnoses, which has good reliability, with kappas ranging from 0.65 to 1.0 [34]. The primary measure of substance use quantity and frequency will be the interviewer-administered Form 90 Substance Use Interview [50]. This interview yields total number of days in the 90 days prior to last use of all alcohol/drug use, total number of drugs used, age at first use, lifetime weeks of use and level of use, and has been used successfully with runaway and homeless youth [51]. As further validation of self-reported drug use, urine toxicology screens (tox-cups) will be collected for youth at baseline, 6- and 12-month follow-up. Urine screens are completed onsite with one-step *BMC ToxCup® Test Kit* (Branan Medical Corp., Irvine, CA), which provides instant readings for the detection of cannabinoids, amphetamines, methamphetamines, phencyclidine (PCP), cocaine/crack, and opiates.

### Secondary outcomes

Secondary outcome measures will be collected in order to evaluate the additional outcomes hypothesized to be positively associated with the housing intervention, including mental and physical health outcomes, additional measures of alcohol and other drug use, housing stability, employment or education, and HIV risk. The percent of days of other alcohol/drug use will measured based on the Form 90 (described above), as will measures of housing and employment (e.g. percent of days housed, percent of days employed, percent of days in school/training). Drug use consequences will be measured using the Shortened Inventory of Problems—Alcohol and Drugs (SIP-AD) [52]. The HIV risk behaviors measured will include condom use, intravenous drug use, history of STDs, and sexual activity with multiple and/or high risk partners [53, 54]. The survey items measuring these behaviors are adapted from prior surveys among homeless youth [55]. The responses to individual items are combined to yield a total score ranging from 0 from 7 points, consistent with prior studies [41, 56–58]. Self-regulation will be measured with the Short Self-Regulation Scale, a 31-item instrument of cognitive competence that is highly correlated with the longer 63-item survey (alpha = 0.92) [59, 60]. The Short Self-Regulation Scale assesses youth’s ability to regulate cognitions and behaviors related to achieving their goals. The Beck Depression Inventory II (BDI-II) will be collected to measure mood, cognitive and somatic aspects of depression [61]. Resilience will be measured with the Brief Resilience Scale (BRS), a Likert-type 5-item instrument that measures the ability to bounce back or recover from stress [62]. The BRS is recommended for use in stressful contexts and has shown good internal consistency reliability (0.80–0.91) [62, 63]. Cognitive distortions will be measured with the Cognitive Distortions Questionnaire (CD-QUEST), a 15-item measure of cognitive distortions that assesses the frequency and intensity of common cognitive distortions over the past week [64]. The CD-QUEST has demonstrated good internal consistency (alphas = 0.80–0.91) and convergent validity across a variety of clinical and non-clinical samples [64, 65]. The Coping Inventory for Stressful Situations-Short Form (CISS-SFC) is a 21-item measure that yields 3 subscale scores: Task-, emotion-, and avoidance-oriented coping [66]. Reliability for the three subscales is 0.90, 0.88 and 0.83, respectively [67]. Health status will be assessed with the Short Form-6 is a standardized, internationally used instrument that provides a general measure of health status [68]. Its construct validity has been evaluated specifically with adult users of homeless day shelters [69]. Finally, the client satisfaction questionnaire (CSQ-8) will be collected, which is one of a limited number of standardized satisfaction measures that have been used widely across mental health services and has sound psychometric properties for substance users [70, 71].

### Primary mediators

Mediators were selected based on the biopsychosocial model and Bronfenbrenner’s Ecological Systems model. The current study hypothesizes that increased access to social resources and social support and decreased exposure to violence will promote positive changes in outcomes and that the effect will be strongest for those individuals randomized to receive HF with risk prevention services. Several measures will be collected as possible primary mediators of the treatment effects. First, the number of service contacts will be based on the study’s service contact log. The log identifies the youth’s frequency of contact with several different services (e.g. employment, medical, shelters/drop-ins). Second, the Working Alliance Inventory (WAI) will be used to measure the perceived level of connection and shared goals between the outreach worker and client and yields 3 subscale scores: task agreement, goal agreement, and bond development [72]. Third, the Social Network Inventory (SNI) will be collected as a measure of social support and has been used in multiple studies with homeless populations and high risk adolescents [73–75], with test–retest reliabilities of 0.74 to 0.82 for the key SNI variables [76]. Satisfaction, substance use and illegal behaviors of network members is queried. This study will utilize a support contact measure based on the mean frequency of contact across all network members who the respondent indicated as having provided emotional, tangible, or other support. Finally, number of violence exposures (such as robbery or assault) will be assessed with self-report items from the HEQ.

### Secondary mediators

Secondary mediators are assessed to evaluate whether individual resources are activated by the positive changes in the primary mediators described above. Measures of stress response (self-report and physiological) and self-efficacy will be collected as possible secondary mediators of the treatment effect. Individual stress will be measured using Cohen’s Perceived Stress Scale (PSS) [77], which is one of the most widely used instruments for measuring an individual’s perception of their stress levels and reports both high validity and reliability [78]. The PSS has 10 items, each scored on a scale of 0–4, and the total score ranges from 0 to 56, with higher scores indicating greater perceived stress. We measure the chronic physiologic stress response through cumulative cortisol concentration collected and assayed from hair. Hair grows approximately 1 cm a month, thus each 1 cm of hair growth approximates the mean cortisol level for the corresponding month. We will assay up to 3 cm of hair growth from the scalp at each 3-month data collection time point to capture the mean cortisol concentration over the preceding 3 months. Prior research found interventions can improve salivary cortisol regulation [79], and although limited in number due to the novelty of using hair to measure cortisol, the evidence suggests improvements in hair cortisol regulation as well [80–82]. To collect the hair samples, approximately 10–75 mg (approximate width of shoelace tip when bunched) of hair is cut with professional shears from the posterior vertex region of the scalp as close to the scalp as possible [83, 84]. The hair will be assayed in duplicate for the mean cortisol value at the Ford lab via adapted protocol by Meyer et al. [83]. Finally, self-efficacy will be assessed as a mediator with respect to three health behaviors. First, specific self-efficacy for alcohol and other drug use will be assessed using the 8-item Drug-Taking Confidence Questionnaire-8 (DTCQ-8) [85]. Second, the 9-item HIV Behaviors Self-Efficacy Scale measures specific self-efficacy in regard to HIV risk behaviors [86]. It has shown high internal consistency (alpha = 0.77) and support for its construct validity [86]. Third, personal control self-efficacy will be assessed using Pearlin and Schooler’s 7-item Mastery Scale [87]. The instrument has robust psychometric properties and has proven validity with those experiencing homelessness [87–89].

## Analytic plan

The patterns of missing data will be examined before each analysis. If data are missing at random, they will be estimated using full information maximum likelihood (FIML) or multiple imputations (MI) method. When data are missing completely at random (MCAR) or are missing at random (MAR), both FIML and ML produce unbiased results [90]. If the missingness cannot be explained by observed data, that is, data are Missing Not at Random (MNAR), data analysis will be conducted using the pattern mixture model framework [91].

### Aim 1 analysis

The impact of the two intervention conditions (i.e., 6-months housing + opioid and related risk preventive services and opioid and related risk preventive services alone) on the primary and secondary outcomes will be tested in Aim 1. Specifically, the primary outcome, time to the onset of OUD, will be analyzed using the discrete-time survival analysis. In this analysis, whether the onset of OUD has occurred at a specific time point is included as the indicator of a latent factor, the proportional odds for the hazard of OUD, which is predicted by the contrasts between conditions. Under the proportional hazards assumption, it is expected that youth assigned to the housing + preventive services condition will have longer delay in the onset of OUD than those in the preventive services alone condition. Time varying covariate will be explored if the proportional hazard assumption is violated. For the primary outcome (% days opioid use) and secondary outcomes that are continuous variables, latent growth models (LGM) will be conducted to estimate the trajectories of change across five time points (baseline, 3, 6, 9 and 12 months follow-up) to estimate the trajectories of change over time. Differences between treatment conditions on estimated growth parameters, including intercepts (i.e., initial status) and slopes (i.e., the rates of change), will be tested. It is expected that those assigned to the 6-month housing + opioid and related risk prevention services group will show greater decreases in opioid use (for those using opioids), as well as greater improvement in secondary outcomes, and that these improvements will maintain for a longer period of time, compared to those assigned to prevention services alone. To increase validity of the conclusions drawn from the analysis, the number of intervention sessions and contacts with other service providers will be covariates in the LGM analyses.

### Aim 2 analysis

To test the proposed behavioral change mechanisms (i.e., social and individual resources), a series of path analysis will be conducted. Specifically, we will first test whether the primary mediator (e.g., service connections, social support) or secondary mediator (e.g., self-efficacy, stress) assessed at the 3- or 6-month follow-up mediates the association between intervention and outcome at 6-month and 9-/12-month follow-up, respectively. We expect that the intervention condition with the housing component will produce greater improvement in social/individual resources, which in turn will predict better outcomes. We will also estimate serial mediation models, in which both primary and secondary mediators are included, to test whether intervention first affects social resources, which then lead to changes in individual resources and subsequent outcomes. In these models, the baseline assessment of the mediators and outcomes will be controlled for in the analysis. Following MacKinnon and colleagues [92], the product of the coefficient of the path from the independent variable to the mediator(s) and the coefficient of the path from the mediator(s) to the outcomes will be computed as the indirect (mediation) effect between the intervention and outcomes. The strength and significance of the mediation will be estimated using a bootstrap sampling method [93].

### Statistical power analysis

The power analyses were conducted using the Monte Carlo simulation method. The MPI’s ongoing study testing the effect of housing support on homeless mothers reveals medium effect sizes in reducing substance use (*d* = 0.49) and depressive symptoms (*d* = 0.59), and a large effect (*d* = 1.26) in improving housing stability, favoring the housing intervention over TAU/assessment only. Given that the proposed study tests housing support against an active intervention, and the differences between the two conditions may be smaller than that between housing support and TAU, we assumed small-to-medium effect sizes when conducting the power analysis. For the latent growth analyses, with dichotomous predictors (contrasts between intervention conditions) that have regression coefficients of 0.15 (small-to-medium effect size) for the slopes of growth factors [94], a sample size of 240, with an overall attrition rate of 15%, can produce a power of 0.86 to detect the intervention effect on the growth rate of outcomes and a power of 0.94 for the survival analysis of OUD. For Aim 2, in the simulated mediation analysis, we again assumed a small-to-medium effect size for the intervention effect. Data from the MPI’s ongoing clinical trial show that the associations between social/individual resources and outcomes are of medium effect size or larger (e.g., personal control is associated with depressive symptoms at *r* = -0.46 and with general mental health at *r* = 0.43). Thus, we assumed medium effect sizes for the mediator to outcome pathways. Following the model specification suggested by Thoemmes et al. [95], the proposed sample size could provide a power of 0.93 to detect mediating effects for the one mediator model and a power of 0.82 for the serial mediation model with two mediators.

### Economic analysis

We will quantify the economic cost of both interventions, the housing + prevention services and prevention services alone. The cost comparison will lead to a cost-effectiveness analysis of the added benefit of housing. Resources used and their associated dollar cost are collected using activity–based costing method [96]. The steps for conducting cost analysis include (i) inventorying resources consumed, (ii) counting the number of units consumed in each resource category, (iii) estimating cost per unit of each resource type, and (iv) calculating total costs of the intervention and expressing this cost per-youth. Pre-implementation and implementation costs will be collected separately since the implementation strategies used during preparation phases are different than the implementation strategies used once an intervention is deployed [97]. Pre-implementation strategies include planning meetings, training of advocates, hiring, development of policies and procedures, and management. We will count the number of units used via weekly time sheets and other entries. Implementation activities include time spent recruiting participants, screening for eligibility, time of trained professionals delivering housing delivering SBOA, supervision, monthly housing and utilities, and other resources including mileage and office supplies. Time spent for each of these activities are recorded via Qualtrics survey. Housing and utilities are monthly recorded on an excel sheet. For the effectiveness analysis, the change in initiation of opioid use between assessments, as well as the change in escalation of opioid use, will be utilized.

## Discussion

Reviews conducted on research with homeless youth found mostly small samples that focus on a variety of disparate outcomes, but none that test comprehensive, multi-component prevention interventions [5, 98]. Such research is essential to move the field forward by providing a practical and evidence-based guide to programs and governmental entities that seek to assist these youth. To our knowledge, this is the first trial to test Housing First for homeless youth. Research shows that the majority of homeless youths do not access resources meant for them, including shelters [99]. Although shelters are the primary avenue for exiting street life, alternatives that work for those who refuse to access shelters, and for communities where shelters are not available, are needed. Therefore, if successful, the HOME intervention has the potential to generalize to those communities. Also, physiological measures of stress associated with physical and mental health have primarily been obtained using cross-sectional designs [100, 101]. Obtaining physiological measures of stress longitudinally is rare and allows us to examine individual variation of stress as a mediating response to housing and prevention services. Finally, this study has unique practice implications because it addresses a critical public health problem of opioid use disorders among youth. The cost analysis will provide essential information for community adoption of the intervention. Many states require the use of evidence-based practice by grantees, and several cities have launched campaigns to address the opioid epidemic which puts pressure on systems to find interventions that work. Interventions focused on preventing opioid use and ending substance use, homelessness and its associated problems need to be a top priority of local, state and federal governments, as well as of communities. This study protocol, and upcoming study findings, are initial steps in addressing these needs.

## Data Availability

Not applicable.

## References

[1] Compton WM, Volkow ND (2006). Major increases in opioid analgesic abuse in the United States: concerns and strategies. Drug Alcohol Depend.

[2] DeBeck K, Kerr T, Nolan S, Dong H, Montaner J, Wood E (2016). Inability to access addiction treatment predicts injection initiation among street-involved youth in a Canadian setting. Subst Abuse Treat Prev Policy.

[3] Brands B, Leslie K, Catz-Biro L, Li S (2005). Heroin use and barriers to treatment in street-involved youth. Addict Res Theory.

[4] Goldman-Hasbun J, Kerr T, Nosova E, Shulha H, Wood E, DeBeck K (2019). Initiation into heroin use among street-involved youth in a Canadian setting: a longitudinal cohort study. Drug Alcohol Depend.

[5] Edidin JP, Ganim Z, Hunter SJ, Karnik NS (2012). The mental and physical health of homeless youth: a literature review. Child Psychiatry Hum Dev.

[6] Morton MH, Dworsky A, Matjasko JL, Curry SR, Schlueter D, Chavez R (2018). Prevalence and correlates of youth homelessness in the United States. J Adolesc Health.

[7] Rhoades H, Winetrobe H, Rice E (2014). Prescription drug misuse among homeless youth. Drug Alcohol Depend.

[8] Cheng T, Small W, Dong H, Nosova E, Hayashi K, DeBeck K (2018). An age-based analysis of nonmedical prescription opioid use among people who use illegal drugs in Vancouver, Canada. Subst Abuse Treat Prev Policy.

[9] Goldman-Hasbun J, DeBeck K, Buxton JA, Nosova E, Wood E, Kerr T (2017). Knowledge and possession of take-home naloxone kits among street-involved youth in a Canadian setting: a cohort study. Harm Reduct J.

[10] Braciszewski JM, Stout RL (2012). Substance use among current and former foster youth: a systematic review. Child Youth Serv Rev.

[11] Gaetz S (2004). Safe streets for whom? Homeless youth, social exclusion, and criminal victimization. Can J Criminol Crim.

[12] Goering P, Wasylenki D, Lindsay S, Lemire D, Rhodes A (1997). Process and outcome in a hostel outreach program for homeless clients with severe mental illness. Am J Orthopsychiat.

[13] Walker A, Walker C (1997). Britain divided: the growth of social exclusion in the 1980s and 1990s.

[14] Dworsky AL, Piliavin I (2000). Homeless spell exits and returns: Substantive and methodological elaborations on recent studies. Soc Serv Rev.

[15] Zlotnick C, Tam T, Robertson MJ (2003). Disaffiliation, substance use, and exiting homelessness. Subst Use Misuse.

[16] Slesnick N, Kang MJ, Bonomi AE, Prestopnik JL (2008). Six- and twelve-month outcomes among homeless youth accessing therapy and case management services through an urban drop-in center. Health Serv Res.

[17] Faggiano F, Minozzi S, Versino E, Buscemi D (2014). Universal school-based prevention for illicit drug use. Cochrane Db Syst Rev..

[18] Kumpfer KL, Hansen W, Scheier L, Hansen W (2014). Parenting and teen drug use: the most recent findings from research, prevention, and treatment. Family based prevention programs.

[19] Strang J, Babor T, Caulkins J, Fischer B, Foxcroft D, Humphreys K (2012). Drug policy and the public good: evidence for effective interventions. Lancet.

[20] Aidala A, Cross JE, Stall R, Harre D, Sumartojo E (2005). Housing status and HIV risk behaviors: implications for prevention and policy. AIDS Behav.

[21] Cappelletti ER, Kreuter MW, Boyum S, Thompson T (2015). Basic needs, stress and the effects of tailored health communication in vulnerable populations. Health Educ Res.

[22] National Alliance to End Homelessness. Ending Chronic Homelessness Saves Taxpayers Money http://endhomelessness.org/wp-content/uploads/2017/06/Cost-Savings-from-PSH.pdf.

[23] Florence CS, Zhou C, Luo F, Xu L (2016). The economic burden of prescription opioid overdose, abuse, and dependence in the United States, 2013. Med Care.

[24] Gaetz S, Dej, E.,. A New Direction: A Framework for Homeless Prevention: Canadian Observatory on Homelessness; 2017. https://www.homelesshub.ca/ANewDirection.

[25] Goering PN, Streiner DL (2015). Putting housing first: the evidence and impact. Can J Psychiat.

[26] Tsemberis S (1999). From streets to homes: An innovative approach to supported housing for homeless adults with psychiatric disabilities. J Community Psychol.

[27] Tsemberis S, Gulcur L, Nakae M (2004). Housing First, consumer choice, and harm reduction for homeless individuals with a dual diagnosis. Am J Public Health.

[28] Padgett DK, Stanhope V, Henwood BF, Stefancic A (2011). Substance use outcomes among homeless clients with serious mental illness: comparing housing first with treatment first programs. Community Ment Hlt J.

[29] Sadowski LS, Kee RA, VanderWeele TJ, Buchanan D (2009). Effect of a housing and case management program on emergency department visits and hospitalizations among chronically ill homeless adults: a randomized trial. JAMA.

[30] Collins SE, Malone DK, Clifasefi SL, Ginzler JA, Garner MD, Burlingham B (2012). Project-based Housing First for chronically homeless individuals with alcohol problems: within-subjects analyses of 2-year alcohol trajectories. Am J Public Health.

[31] Leff HS, Chow CM, Pepin R, Conley J, Allen IE, Seaman CA (2009). Does one size fit all? What we can and can't learn from a meta-analysis of housing models for persons with mental illness. Psychiat Serv.

[32] Gaetz S, Gulliver-Garcia T, Richter T (2014). The State of Homelessness in Canada: 2014.

[33] McKinney-Vento Homeless Assistance Act, Re-Authorized (2002), 11431 et seq 725.

[34] First MB, Williams J.B.W., Karg, R.S., & Spitzer, R.L, . User’s guide for the Structured Clinical Interview for DSM-5 Disorders: Research version. Arlington, VA: American Psychological Association; 2015.

[35] United States Interagency Council on Homelessness. Opening Doors: Federal Strategic Plan to Prevent and End Homelessness 2010. https://www.usich.gov/resources/uploads/asset_library/USICH_OD_Amendment_WEB_091112v2.pdf.

[36] Spoth RL, Greenberg MT (2005). Toward a comprehensive strategy for effective practitioner-scientist partnerships and larger-scale community health and well-being. Am J Community Psychol.

[37] Baer JS, Ginzler JA, Peterson PL (2003). DSM-IV alcohol and substance abuse and dependence in homeless youth. J Stud Alcohol.

[38] Baldwin SA, Murray DM, Shadish WR, Pals SL, Holland JM, Abramowitz JS (2011). Intraclass correlation associated with therapists: estimates and applications in planning psychotherapy research. Cogn Behav Ther.

[39] Miller WR, Rollnick S (2012). Motivational Interviewing: Helping people change.

[40] Raleigh-duroff C (2004). Factors that influence homeless adolescents to leave or stay living on the street. Child Adolesc Soc Work J.

[41] Carmona J, Slesnick N, Guo X, Letcher A (2014). Reducing high risk behaviors among street living youth: outcomes of an integrated prevention intervention. Child Youth Serv Rev.

[42] Slesnick N, Kang MJ (2008). The impact of an integrated treatment on HIV risk behavior among homeless youth: a randomized controlled trial. J Behav Med.

[43] Copeland L, McNamara R, Kelson M, Simpson S (2015). Mechanisms of change within motivational interviewing in relation to health behaviors outcomes: a systematic review. Patient Educ Couns.

[44] Baer JS, Peterson PL, Wells EA (2004). Rationale and design of a brief substance use intervention for homeless adolescents. Addict Res Theory.

[45] Slesnick N, Erdem G (2013). Efficacy of ecologically-based treatment with substance-abusing homeless mothers: substance use and housing outcomes. J Subst Abuse Treat.

[46] Substance Abuse and Mental Health Services Administration (2019). Enhancing motivation for change in substance use disorder treatment. Treatment Improvement Protocol (TIP) Series No. 35.

[47] Slesnick N, Erdem G (2012). Intervention for homeless, substance abusing mothers: findings from a non-randomized pilot. Behav Med.

[48] Guo X, Slesnick N, Feng X (2016). Housing and support services with homeless mothers: benefits to the mother and her children. Community Ment Health J.

[49] Slesnick N, Guo X, Brakenhoff B, Bantchevska D (2015). A comparison of three interventions for homeless youth evidencing substance use disorders: results of a randomized clinical trial. J Subst Abuse Treat.

[50] Miller W (1996). Form 90 a structured assessment interview for drinking and related problem behaviors. Project MATCH Monograph Seriece, 5.

[51] Slesnick N, Tonigan JS (2004). Assessment of alcohol and other drug use by runaway youths: a test-retest study of the Form 90. Alcohol Treat Q.

[52] Gillespie W, Holt JL, Blackwell RL (2007). Measuring outcomes of alcohol, marijuana, and cocaine use among college students: a preliminary test of the shortened inventory of problems-alcohol and drugs (SIP-AD). J Drug Issues.

[53] De Rosa CJ, Montgomery SB, Hyde J, Iverson E, Kipke MD (2001). HIV risk behavior and HIV testing: a comparison of rates and associated factors among homeless and runaway adolescents in two cities. AIDS Educ Prev.

[54] Garofalo R, Deleon J, Osmer E, Doll M, Harper GW (2006). Overlooked, misunderstood and at-risk: exploring the lives and HIV risk of ethnic minority male-to-female transgender youth. J Adolesc Health.

[55] Johnson TP, Aschkenasy JR, Herbers MR, Gillenwater SA (1996). Self-reported risk factors for AIDS among homeless youth. AIDS Educ Prev.

[56] Collins J, Slesnick N (2011). Factors associated with motivation to change HIV risk and substance use behaviors among homeless youth. J Soc Work Pract Addict.

[57] Gangamma R, Slesnick N, Toviessi P, Serovich J (2008). Comparison of HIV risks among gay, lesbian, bisexual and heterosexual homeless youth. J Youth Adolesc.

[58] Slesnick N, Bartle-Haring S, Glebova T, Glade AC (2006). Homeless adolescent parents: HIV risk, family structure and individual problem behaviors. J Adolesc Health.

[59] Brown JM, Miller WR, Lawendowski LA, Jackson LVTL (1999). The Self-Regulation Questionnaire. Innovations in clinical practice: a sourcebook Sarasota.

[60] Carey KB, Neal DJ, Collins SE (2004). A psychometric analysis of the self-regulation questionnaire. Addict Behav.

[61] Beck AT, Erbaugh J, Ward CH, Mock J, Mendelsohn M (1961). An inventory for measuring depression. Arch Gen Psychiat.

[62] Smith BW, Dalen J, Wiggins K, Tooley E, Christopher P, Bernard J (2008). The brief resilience scale: assessing the ability to bounce back. Int J Behav Med.

[63] Windle G, Bennett KM, Noyes J (2011). A methodological review of resilience measurement scales. Health Qual Life Outcomes.

[64] de Oliveira IR, Seixas C, Osorio FL, Crippa JA, de Abreu JN, Menezes IG (2015). Evaluation of the psychometric properties of the cognitive distortions questionnaire (CD-Quest) in a sample of undergraduate students. Innov Clin Neurosci.

[65] Batmaz S, Kocbiyik S, Yalcinkaya-Alkar O, Turkcapar MH (2016). Cognitive distortions mediate the relationship between defense styles and depression in female outpatients. Eur J Psychiat.

[66] Cohan SL, Jang KL, Stein MB (2006). Confirmatory factor analysis of a short form of the Coping Inventory for Stressful Situations. J Clin Psychol.

[67] Parker JDA, Endler NS (1992). Coping with coping assessment - a critical-review. Eur J Personality.

[68] Ware J, Kosinski M, Keller SD (1996). A 12-Item Short-Form Health Survey: construction of scales and preliminary tests of reliability and validity. Med Care.

[69] Larson CO (2002). Use of the SF-12 instrument for measuring the health of homeless persons. Health Serv Res.

[70] Larsen DL, Attkisson CC, Hargreaves WA, Nguyen TD (1979). Assessment of client/patient satisfaction: development of a general scale. Eval Program Plann.

[71] Kelly PJ, Kyngdon F, Ingram I, Deane FP, Baker AL, Osborne BA (2018). The client satisfaction questionnaire-8: psychometric properties in a cross-sectional survey of people attending residential substance abuse treatment. Drug Alcohol Rev.

[72] Horvath AO, Greenberg LS (1989). Development and validation of the working alliance inventory. J Couns Psychol.

[73] Stein CH, Rappaport J, Seidman E (1995). Assessing the social networks of people with psychiatric disability from multiple perspectives. Community Ment Hlt J.

[74] Urberg K, Goldstein MS, Toro PA (2005). Supportive relationships as a moderator of the effects of parent and peer drinking on adolescent drinking. J Res Adolescence.

[75] Toro PA, Hobden KL, Durham KW, Oko-Riebau M, Bokszczanin A (2014). Comparing the characteristics of homeless adults in Poland and the United States. Am J Community Psychol.

[76] Bates DS, Toro PA (1999). Developing measures to assess social support among homeless and poor people. J Community Psychol.

[77] Cohen S, Kamarck T, Mermelstein R (1983). A global measure of perceived stress. J Health Soc Behav.

[78] Cohen S, Williamson G (1988). Perceived stress in a probability sample of the US.

[79] Slopen N, McLaughlin KA, Shonkoff JP (2014). Interventions to improve cortisol regulation in children: a systematic review. Pediatrics.

[80] Dajani R, Hadfield K, van Uum S, Greff M, Panter-Brick C (2018). Hair cortisol concentrations in war-affected adolescents: A prospective intervention trial. Psychoneuroendocrinology.

[81] Charalampopoulou M, Bacopoulou F, Syrigos KN, Filopoulos E, Chrousos GP, Darviri C (2020). The effects of pythagorean self-awareness intervention on breast cancer patients undergoing adjuvant therapy: a pilot randomized controlled trial. Breast.

[82] Goldberg SB, Manley AR, Smith SS, Greeson JM, Russell E, Van Uum S (2014). Hair cortisol as a biomarker of stress in mindfulness training for smokers. J Altern Complem Med.

[83] Meyer J, Novak M, Hamel A, Rosenberg K (2014). Extraction and analysis of cortisol from human and monkey hair. J Vis Exp.

[84] Russell E, Koren G, Rieder M, Van Uum S (2012). Hair cortisol as a biological marker of chronic stress: current status, future directions and unanswered questions. Psychoneuroendocrinology.

[85] Sklar SM, Turner NE (1999). A brief measure for the assessment of coping self-efficacy among alcohol and other drug users. Addiction.

[86] Smith KW, McGraw SA, Costa LA, McKinlay JB (1996). A self-efficacy scale for HIV risk behaviors: development and evaluation. AIDS Educ Prev.

[87] Pearlin LI, Schooler C (1978). The structure of coping. J Health Soc Behav.

[88] Greenwood RM, Schaefer-McDaniel NJ, Winkel G, Tsemberis SJ (2005). Decreasing psychiatric symptoms by increasing choice in services for adults with histories of homelessness. Am J Community Psychol.

[89] Shern DL, Tsemberis S, Anthony W, Lovell AM, Richmond L, Felton CJ (2000). Serving street-dwelling individuals with psychiatric disabilities: outcomes of a psychiatric rehabilitation clinical trial. Am J Public Health.

[90] Enders CK (2010). Applied missing data analysis.

[91] Roy J (2003). Modeling longitudinal data with nonignorable dropouts using a latent dropout class model. Biometrics.

[92] Mackinnon DP (2008). Introduction to Statistical Mediation Analysis.

[93] Shrout PE, Bolger N (2002). Mediation in experimental and nonexperimental studies: new procedures and recommendations. Psychol Methods.

[94] Muthen LK, Muthen BO (2002). How to use a Monte Carlo study to decide on sample size and determine power. Struct Equ Modeling.

[95] Thoemmes F, MacKinnon DP, Reiser MR (2010). Power Analysis for Complex Mediational Designs Using Monte Carlo Methods. Structural Equation Modeling-a Multidisciplinary Journal.

[96] Neumann P, Sanders GD, Russell LB, Siegel JE, Ganiats TG (2017). Cost-Effectiveness in Health and Medicine.

[97] Bunger AC, Powell BJ, Robertson HA, MacDowell H, Birken SA, Shea C (2017). Tracking implementation strategies: a description of a practical approach and early findings. Health Res Policy Syst.

[98] Slesnick N, Dashora P, Letcher A, Erdem G, Serovich J (2009). A review of services and interventions for runaway and homeless youth: moving forward. Child Youth Serv Rev.

[99] Kelly K, Caputo T (2007). Health and street/homeless youth. J Health Psychol.

[100] Abraham SB, Rubino D, Sinaii N, Ramsey S, Nieman LK (2013). Cortisol, obesity, and the metabolic syndrome: a cross-sectional study of obese subjects and review of the literature. Obesity.

[101] Veldhorst MAB, Noppe G, Jongejan MHTM, Kok CBM, Mekic S, Koper JW (2014). Increased scalp hair cortisol concentrations in obese children. J Clin Endocr Metab.

